# Metabolic adjustment upon repetitive substrate perturbations using dynamic ^13^C-tracing in yeast

**DOI:** 10.1186/s12934-017-0778-6

**Published:** 2017-09-25

**Authors:** C. A. Suarez-Mendez, C. Ras, S. A. Wahl

**Affiliations:** 1 Department of Biotechnology, Delft University of Technology, Van der Maasweg, 92629 HZ Delft, The Netherlands; 2 Kluyver Centre for Genomics of Industrial Fermentation, P.O. Box 5057, 2600 GA Delft, The Netherlands; 3 Present Address: Department of Processes and Energy, Universidad Nacional de Colombia, Carrera 80 No. 65-223, Medellin, Colombia

**Keywords:** Dynamic fluxes, ^13^C labeling, Multiple perturbations, Yeast, Storage carbohydrates, Systems biology, Metabolomics

## Abstract

**Background:**

Natural and industrial environments are dynamic with respect to substrate availability and other conditions like temperature and pH. Especially, metabolism is strongly affected by changes in the extracellular space. Here we study the dynamic flux of central carbon metabolism and storage carbohydrate metabolism under dynamic feast/famine conditions in *Saccharomyces cerevisiae*.

**Results:**

The metabolic flux reacts fast and sensitive to cyclic perturbations in substrate availability. Compared to well-documented stimulus–response experiments using substrate pulses, different metabolic responses are observed. Especially, cells experiencing cyclic perturbations do not show a drop in ATP with the addition of glucose, but an immediate increase in energy charge. Although a high glycolytic flux of up to 5.4 mmol g_DW_^−1^ h^−1^ is observed, no overflow metabolites are detected. From famine to feast the glucose uptake rate increased from 170 to 4788 μmol g_DW_^−1^ h^−1^ in 24 s. Intracellularly, even more drastic changes were observed. Especially, the T6P synthesis rate increased more than 100-fold upon glucose addition. This response indicates that the storage metabolism is very sensitive to changes in glycolytic flux and counterbalances these rapid changes by diverting flux into large pools to prevent substrate accelerated death and potentially refill the central metabolism when substrates become scarce. Using ^13^C-tracer we found a dilution in the labeling of extracellular glucose, G6P, T6P and other metabolites, indicating an influx of unlabeled carbon. It is shown that glycogen and trehalose degradation via different routes could explain these observations. Based on the ^13^C labeling in average 15% of the carbon inflow is recycled via trehalose and glycogen. This average fraction is comparable to the steady-state turnover, but changes significantly during the cycle, indicating the relevance for dynamic regulation of the metabolic flux.

**Conclusions:**

Comparable to electric energy grids, metabolism seems to use storage units to buffer peaks and keep reserves to maintain a robust function. During the applied fast feast/famine conditions about 15% of the metabolized carbon were recycled in storage metabolism. Additionally, the resources were distributed different to steady-state conditions. Most remarkably is a fivefold increased flux towards PPP that generated a reversed flux of transaldolase and the F6P-producing transketolase reactions. Combined with slight changes in the biomass composition, the yield decrease of 5% can be explained.

## Background

Systems biology pursues the understanding of biological systems by unraveling their structure and dynamics [1]. In particular, understanding the function of a metabolic network requires a quantitative description of the network rates and interactions with the help of mathematical models [2]. To generate these models, the system, especially the metabolic concentrations and fluxes have to be observed from a quantitative perspective under different dynamic conditions [3]. Under rapid dynamic environmental conditions strong metabolic flux changes may be expected as these highly depend on the extracellular substrate concentrations. [4] postulated that changing environments demand for adaptability, and cells need to invest in adaptability speed to compete and survive.

It has been observed that storage carbohydrates react sensitive and fast to changes in extracellular glucose concentration [5]. Several authors have proposed that the trehalose cycle acts as an important regulator of the glycolytic flux [6, 7]. In addition, during steady-state ^13^C wash-in experiments a bias in the labeling enrichment of glycolytic intermediates was observed, caused by a transient influx of ^12^C into glycolysis [5]. Recently, it was shown that storage recycling is highly growth-rate dependent [5, 8]. Hence, recycling during dynamic conditions is expected to increase, as these conditions require buffering between high and low substrate conditions.

To study the dynamic interaction of trehalose and other storage intermediates with the central carbon metabolism, intracellular flux estimation is required. An interesting approach consists in collecting time-series of metabolic concentration measurements from dynamic experiments to describe the dynamic response of a pathway to a particular stimulus [9, 10] compared stimulus–response strategies aiming at the identification of in vivo enzyme kinetics. They suggested that only concentration measurements lead to a limited identifiability of the kinetic parameters. To overcome this limitation, the authors suggested that a ^13^C-labeling experiment where the system is at both, metabolic and isotopic non-stationary state, would significantly increase the accuracy of the kinetic parameter estimation. Recently, Abate et al. [11] have proposed a hybrid approach that is based on piece wise affine (PWA) functions in order to estimate fluxes under such dynamic conditions.

Here, dynamic ^13^C tracer experiments are performed to quantify the short-term in- and outflows of the large intracellular trehalose pool as well as central carbon metabolism. The concentration of glycolytic and PPP metabolites as well as enrichment dynamics are monitored over several cycles and a piecewise affine flux approximation in time is used to quantify a dynamic flux profile. Since we focus on the short-term response by monitoring the metabolic activity (intracellular concentrations and fluxes) during cycles of 400 s, it is assumed that the metabolic flux is mainly controlled by metabolic interactions.

## Methods

### Strain and culture conditions

In this work, the haploid yeast *Saccharomyces cerevisiae* CEN PK 113-7D (*Centraalbureau van Schimmelcultures*—Fungal Biodiversity Centre, Utrecht, The Netherlands) was used. Cells from one cryovial (glycerol at −80 °C) were used as seed culture and incubated (30 °C, 200 rpm) for 10 h in 1 L-Erlenmeyer flasks containing 100 mL of minimal medium that contained 13.6 g L^−1^ glucose. The pre-culture was used to inoculate a 7 L bioreactor (Applikon Biotechnology, Delft—The Netherlands) with a working volume of 3.894 L. A low-salt Verduyn minimal medium [12] with a glucose concentration of 7.5 g L^−1^ was used. No ethanol was added to the medium because oscillations were not observed during cultivation. The broth was sparged with pressurized air at 0.992 L min^−1^ (approx. 0.25 vvm) and the vessel was maintained at an overpressure of 0.3 bar. The pH of the broth was controlled at 5.0 by adding either 4 M KOH or 2 M H_2_SO_4_. Temperature and agitation were kept at 30 °C and 600 rpm, respectively, during the complete cultivation. The culture was starved for 2 h after the end of batch phase, which was determined by a strong and fast CO_2_ decrease and dissolved oxygen (DO) increase to almost saturation. The chemostat phase at a dilution rate of 0.1 h^−1^ was started and run for about 5 residence times. At this time, the culture was assumed to be at steady-state as indicated by a constant CO_2_ and O_2_ concentration in the off-gas (1.51 ± 0.01 and 19.6 ± 0.1%, respectively), and a constant biomass density (3.64 ± 0.05 g_DW_ L^−1^).

### Feast famine and labeling setup

**Fig. 1:**
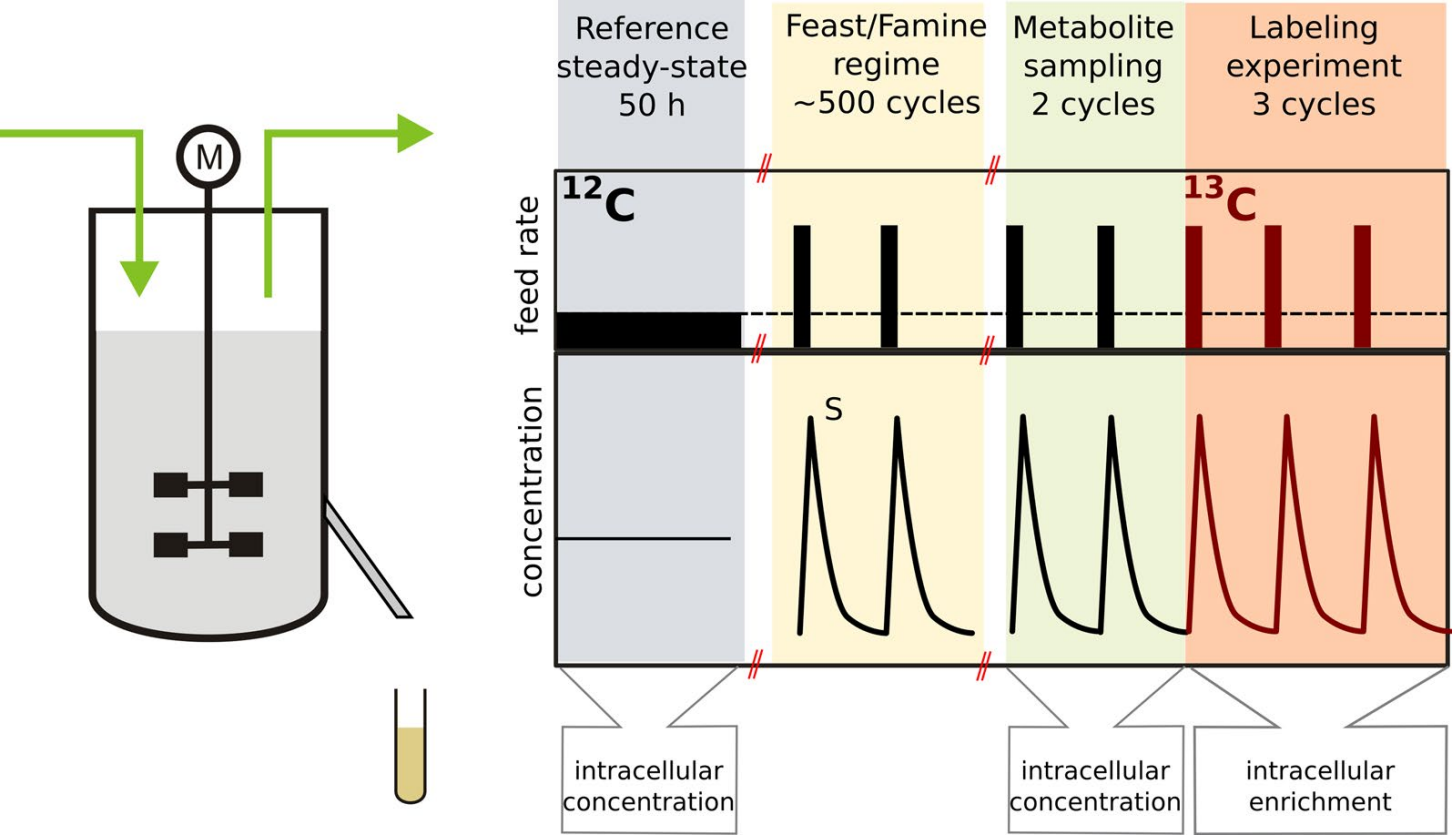
Profile of the experimental feeding and sampling regime. After a chemostat phase (reference steady-state), a block-wise feed is applied at the same average substrate supply and dilution rate. Intracellular concentrations are measured by sampling two cycles, then the feed is switched to labeled substrate and the enrichment is monitored for three consecutive cycles

After 5.3 residence times of running in chemostat mode, the continuous feed was changed to a block-wise regime to start the feast/famine cultivation (Fig. 1). In order to keep an average dilution rate of 0.1 h^−1^, the same amount of fresh medium was added (i.e., 43 mL in 400 s), but block-wise. The feed was switched on for 20 s under control of an automatic timer (PTC-1A, Omega Engineering Inc, USA) feeding 43 mL. In the following 380 s there was no feed addition to complete a total cycle time of 400 s. To maintain the broth volume constant (max. 3.894 + 0.043 L), weight control was used, i.e., 43 mL were withdrawn but during the first 260 s at a flow rate of 0.166 ± 0.001 mL s^−1^.

Initially, unlabeled glucose was used as substrate during the feast/famine cycles for 50 h. After 5 residence times a repeating dynamic state was reached indicated by stable profiles of CO_2_, O_2_ and DO in consecutive cycles. Samples were taken for determination of metabolite concentrations and/or dry weight (3.46 ± 0.01 g_DW_ L^−1^). Right after the sampling for concentrations, the medium was shifted to perform the labeling experiment. The new medium was formulated to have the same components and concentrations as the base medium but with uniformly ^13^C labeled glucose (Cambridge Isotope Laboratories Inc., USA). The labeled medium feed line was prefilled to avoid any perturbation on the cycle time.

### Acquisition, processing and analysis of samples

Broth samples for the measurement of extracellular and intracellular metabolite concentrations were rapidly withdrawn using two separate sampling ports during three subsequent cycles at defined cycle times (5, 10 s,…etc.) to obtain duplicate measurements. Samples for the measurement of mass isotopomer fractions were withdrawn at comparable cycle times after switching to the labeled feed.

#### Extracellular metabolites

For the measurement of extracellular metabolite concentrations, approximately 1.5 mL of broth were withdrawn into a syringe containing pre-cooled (−20 °C) stainless steel beads (approx. 26 g). The cooled broth (~ 1 °C) was immediately filtered as described by [13]. Extracellular (filtrate) glucose was determined by GC–MS analysis as described by [8]. Ethanol, acetate and glycerol concentrations were determined by HPLC using the protocol of [14]. The biomass concentration (dry weight) was determined by a gravimetrical method using pre-dried and pre-weighed membranes (Supor-450, 0.45 μm, 47 mm, Pall Corporation). Membranes containing the biomass cake from 15 mL of broth were dried at 70 °C for 72 h and cooled to room temperature in a desiccator before weighing again. The O_2_ and CO_2_ volume fractions in the off gas were measured by a combined paramagnetic/infrared NGA2000 analyzer (Rosemount Analytics, CA, USA).

#### Intracellular metabolites

For the measurement of intracellular metabolite concentrations, 1.0 ± 0.05 mg-broth was rapidly withdrawn and quenched in 5 mL cold (−40 °C) pure methanol [15] and processed until extraction using hot ethanol (95 °C) following the procedure described in [8]. The extract containing 100 μL of labelled cell extract was then concentrated by complete evaporation of the ethanol–water mixture under vacuum as described by [16]. The dried residues were resuspended in 500 μL milliQ water and centrifuged at 15,000*g* for 5 min at 1 °C, processed and stored according to [8]. Samples were analyzed by GC–MS [17] and/or LC-MSMS [18, 19]. For determination of mass isotopomers the same procedure used for determining concentrations was followed but without the addition of ^13^C cell extract.

#### Modeling and simulation

The metabolic network used for constructing the stoichiometric model was based on the consensus model reported for yeast [20] but significantly lumped and reduced. The model consists of 29 reactions belonging to glycolysis, pentose phosphate pathway, trehalose and glycogen synthesis and degradation as well as the export and extracellular degradation of trehalose. Dynamic fluxes were estimated as piece-wise linear functions defined in the time domain. The time domains were defined by fitting the concentration data with piece-wise affine functions under constraints [21]. Using this approach, breakpoints were set at 0, 24, 69, 218 and 400 s. Having an initial guess for the (independent) fluxes at each breakpoint, the estimation of fluxes was performed based on least-squares optimization to fit both concentration and labeling enrichment measurements.

## Results

### Dynamics of extracellular glucose concentration and ^13^C-labeling during the feast/famine

**Fig. 2:**
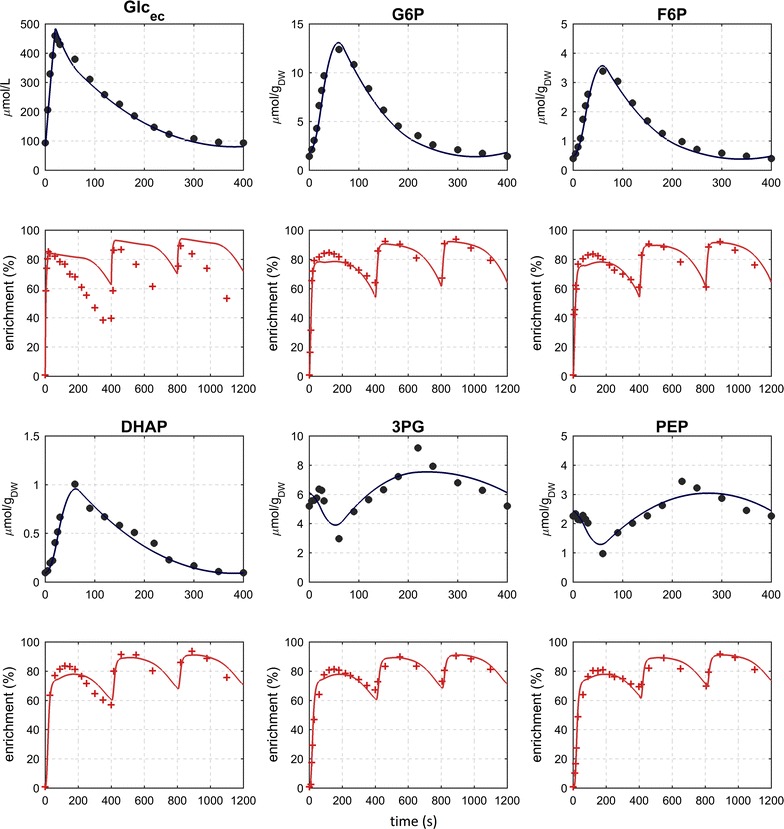
Measured and simulated concentration and ^13^C-labeling enrichment (C-molar average) of glycolytic metabolites during three consecutive feast/famine cycles

Being the entry gate to central metabolism, the dynamics of glucose uptake play a critical role [22]. With the feeding regime used here, the net uptake rate can be easily and accurately determined from the time-series measurements (Fig. 2). The extracellular glucose concentration rapidly increased from 90 ± 3 to 460 ± 6 µM during the first 20 s of the cycle (feeding phase). From 20 s onwards, the glucose concentration decreased in time, basically following a short batch cultivation. Unexpectedly, glucose was not fully consumed at the end of the cycle, which indicates that the cells did not experience severe starvation, but a transient with low substrate availability.

Interestingly, the ^13^C-labeling enrichment showed a decreasing pattern after the feeding phase, indicating a (biological) source of unlabeled glucose. There was no unlabeled glucose added from the feed and putative artifacts from sampling where excluded (i.e. all chemicals and material used was tested for putative remainders of glucose). The dilution in the ^13^C-enrichment of extracellular glucose has also been observed in cultures of *Penicillium chrysogenum* [21]. We hypothesize that storage carbohydrates (trehalose and glycogen) are the source for the observed de-enrichment and we included the synthesis and degradation of these carbohydrates in the network model. Trehalose was also found in the extracellular space, which did not originate from the medium but was produced during the cultivation. The unlabeled glucose can originate from intra- or extracellular degradation of storage materials, both routes were included in the model.

### Dynamics of glycolytic and pentose phosphate pathway metabolites

Metabolites of the upper glycolysis showed a concentration pattern resembling the time-course of extracellular glucose, though with a delay of about 40 s to reach the concentration maximum (i.e. around 60 s after the start of the cycle). The concentration of metabolites in the lower glycolysis exhibited dynamics inverse to upper glycolytic intermediates, which is in agreement with previous findings during pulse perturbations [23]. Comparable to the extracellular glucose enrichment, a decrease in the labeling of glycolytic metabolites was observed, although less prominent.

**Fig. 3:**
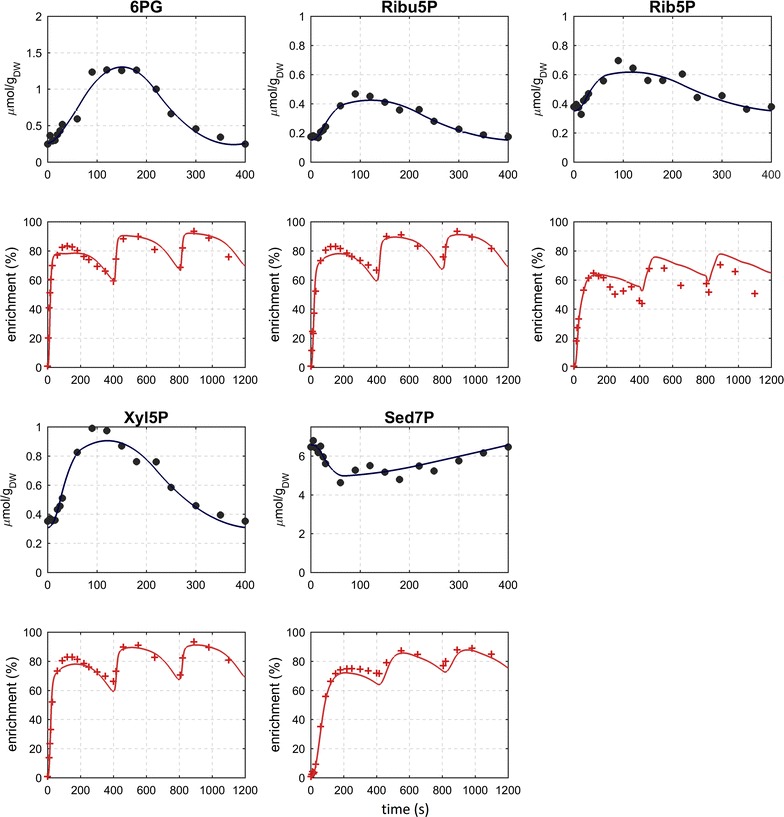
Measured and simulated concentration and ^13^C-labeling enrichment (C-molar average) of PPP metabolites during three consecutive feast/famine cycles

Metabolites of the pentose phosphate pathway showed a response, in both concentration and labeling, that was similar to glycolytic metabolites with the exception of Sed7P (Fig. 3).

### G6P as first branch point in central metabolism

Acting as a first branchpoint, G6P plays an important role in the upper part of the glycolytic pathway, PPP and storage carbohydrate synthesis [24, 25] observed that about two-thirds of the synthesized G6P was used for glycolysis, about 10% was spent towards the pentose phosphate pathway, 20% used for the reversible conversion to G1P while only a small fraction was used for the production of T6P and other pathways. During the feast/famine, the G6P concentration increased about ninefold from 1.43 ± 0.01 μmol _gDW_^−1^ to a maximum at 12.39 ± 0.18 μmol g_DW_^−1^ in 60 s, which confirms the highly dynamic nature of the feast/famine experiment. The concentration and ^13^C-labeling of G6P, F6P and G1P are very comparable, indicating that PGI and PGM operate near equilibrium, i.e. these are bidirectional as reported previously [14, 25]. In contrast to these metabolites the pentose phosphate pathway as well as T6P showed a delay in concentration and enrichment, which will be discussed later.

### Dynamics of the storage carbohydrate intermediates

**Fig. 4:**
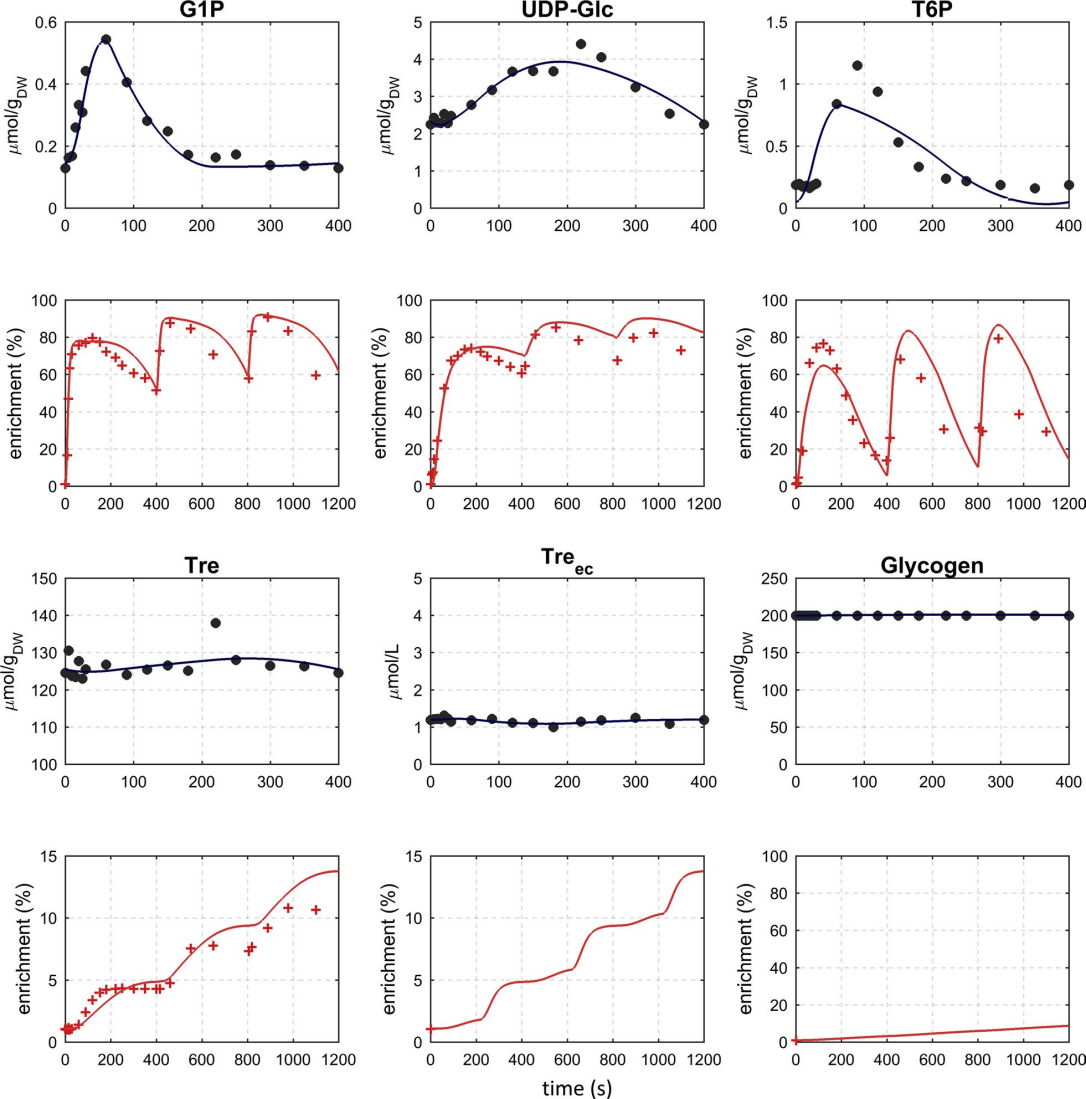
Measured and simulated concentration and ^13^C-labeling enrichment (C-molar average) of metabolites of the storage carbohydrate branches during three consecutive feast/famine cycles. Glycogen and extracellular trehalose ^13^C enrichment was unfortunately not measured

The rapid response of storage carbohydrates has been reported for various dynamic conditions, including even mild perturbations [5]. The metabolites of the storage pathway, T6P, G1P and UDPG showed significant changes in concentration (6-, 4-, 2-fold change respectively). However, while the ^13^C-labeling pattern of G1P and UDPG resembled the G6P labeling, the enrichment profile of T6P showed significant differences, especially a delay of about 40 s and then, after about 100 s, a strong de-enrichment (Fig. 4). This unique behavior in the T6P labeling can only be explained by an influx of unlabeled carbon (potential measurement artifacts like inaccuracy for low concentration were evaluated and can be excluded). In order to reproduce the experimental data, a putative reaction synthesizing T6P from trehalose has been implemented in the metabolic network (putative enzymes are discussed later).

Trehalose showed the highest concentration of all the measured pools (about 126 ± 4 μmol g_DW_^−1^), and the ^13^C-enrichment reached 4.3% at the end of the first cycle (Fig. 4). Remarkably, the trehalose concentration remained nearly constant during the cycle (12% change from minimum to the maximum measurement). The ^13^C-enrichment of trehalose showed a concise increasing pattern, which suggests an active flux through this pool. Especially, ^13^C-influx was observed in the time between 60 and 180 s. After this time, the labeling remained constant until the next feeding phase (i.e., start of a new cycle). The constant labeling enrichment indicates that there was no synthesis during this period (180–400 s). At the same time, there is a decrease in the concentration which is in agreement with the observed entry of low ^13^C-enriched material into glycolysis (G6P).

The observation that trehalose is also present in the extracellular space is an evidence that yeast has an export mechanism for this metabolite (Fig. 4). Cell lysis was excluded because other intracellular metabolites like aminoacids were not detected extracellularly. Trehalose uptake has been shown to depend on the Agt1p transporter [26], which potentially also exports trehalose. In the extracellular space, trehalose is broken down into glucose by the acid trehalase Ath1p. Thermodynamically, this degradation is feasible for the measured concentrations (the equilibrium constant is 115 M at 30 °C [27]).

### Dynamic flux estimation of glycolysis and pentose phosphate pathway

Based on the observed concentrations and labeling measurements, flux values in time were estimated using a piece-wise affine flux modeling approach. The breakpoints were selected based on an optimization of the concentration profiles and represent a compromise to all metabolites [28]. The model can reproduce most features of the experimental data (Figs. 2, 3, 4).

The model can reproduce most of the ^13^C labeling measurements during the three cycles with labeled feed. However, some local deviations between the simulated and experimental labeling can be observed, which originate from (1) suboptimal placement of breakpoints for some metabolites and (2) potentially missing reactions with influence on the labeling patterns. Nevertheless, key features of the measurements are reproduced over the complete three cycles, which increases the confidence that the most important features are included and the calculated fluxes are a reasonable estimation.

**Fig. 5:**
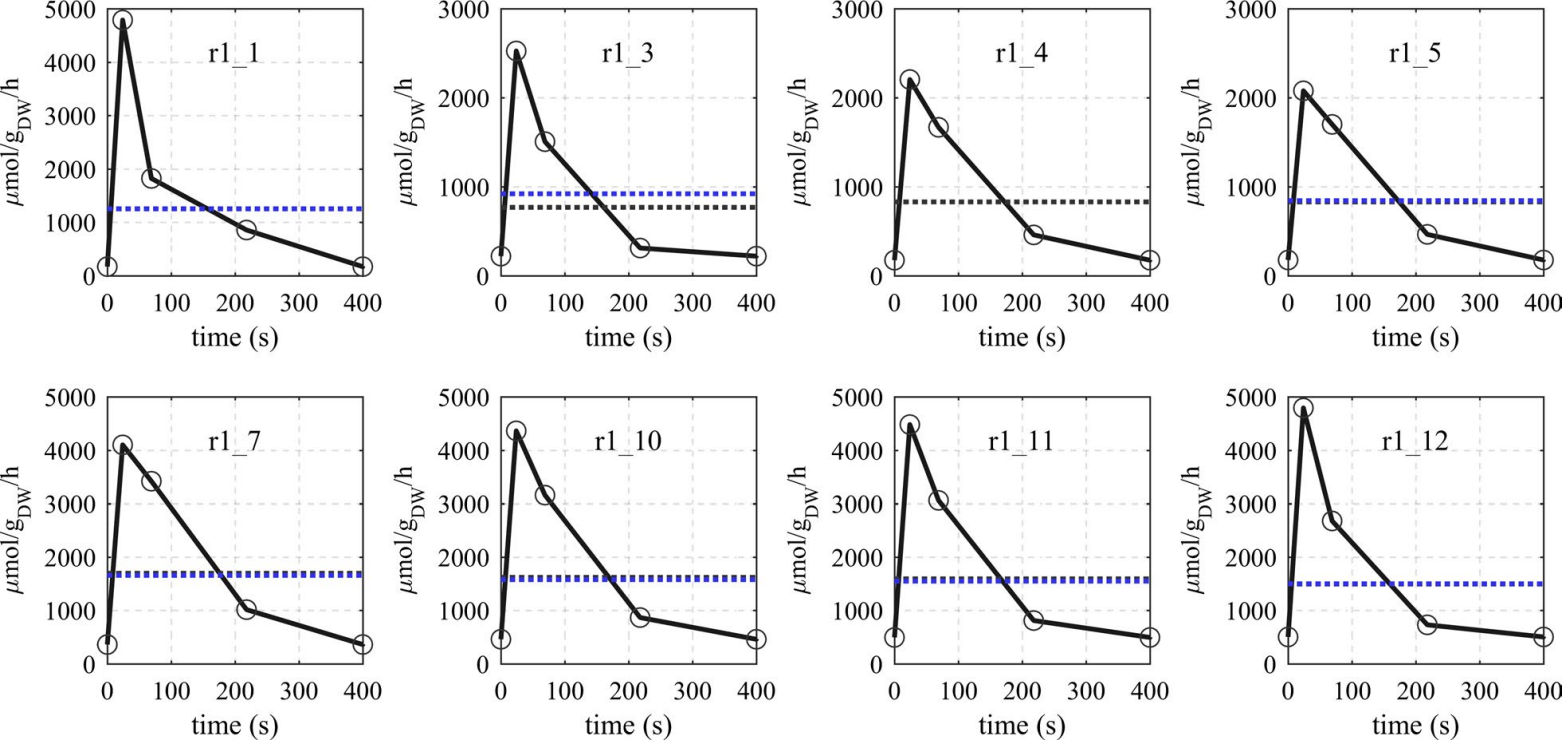
Estimated dynamic fluxes (Glycolysis) during the feast/famine regime. Horizontal lines represent: average flux over the cycle (blue) and steady-state flux a D = 0.1 h^−1^ (black)

Calculations show that the net fluxes in glycolysis followed the pattern of glucose uptake (reaction r1_1) as response to glucose perturbation (Fig. 5). Some glycolytic fluxes changed up to 30-fold during the feeding phase with the glucose uptake increasing from 170 to 4788 μmol g_DW_^−1^ h^−1^ during the first 24 s after the perturbation. The average flux during the cycle, 1253 μmol g_DW_^−1^ h^−1^, was the same as during the steady-state at the corresponding growth rate (1252 μmol g_DW_^−1^ h^−1^ at D = 0.1 h^−1^). This rate is higher than the glucose feeding rate (1156 μmol g_DW_^−1^ h^−1^) which originates from a glucose recycle through the extracellular space as will be discussed later.

The dynamics of the uptake rate mostly follows the extracellular glucose concentration, a strong increase with the onset of the feeding (max of 4788 µmol g-_DW_^−1^ h^−1^), a steep decrease between 24 and 69 s and then further decreasing to the value at the end/beginning of the cycle, 171 µmol g-_DW_^−1^ h^−1^. The downstream glycolytic reactions (r1_3, r1_4, r1_5) resembled the uptake profile with flux values that were about two-thirds of the glucose uptake rate. In addition, with each step the flux profile became smoother, i.e. lower maximum peaks and slower decrease indicating a slight buffering effect of the intermediates.

**Fig. 6:**
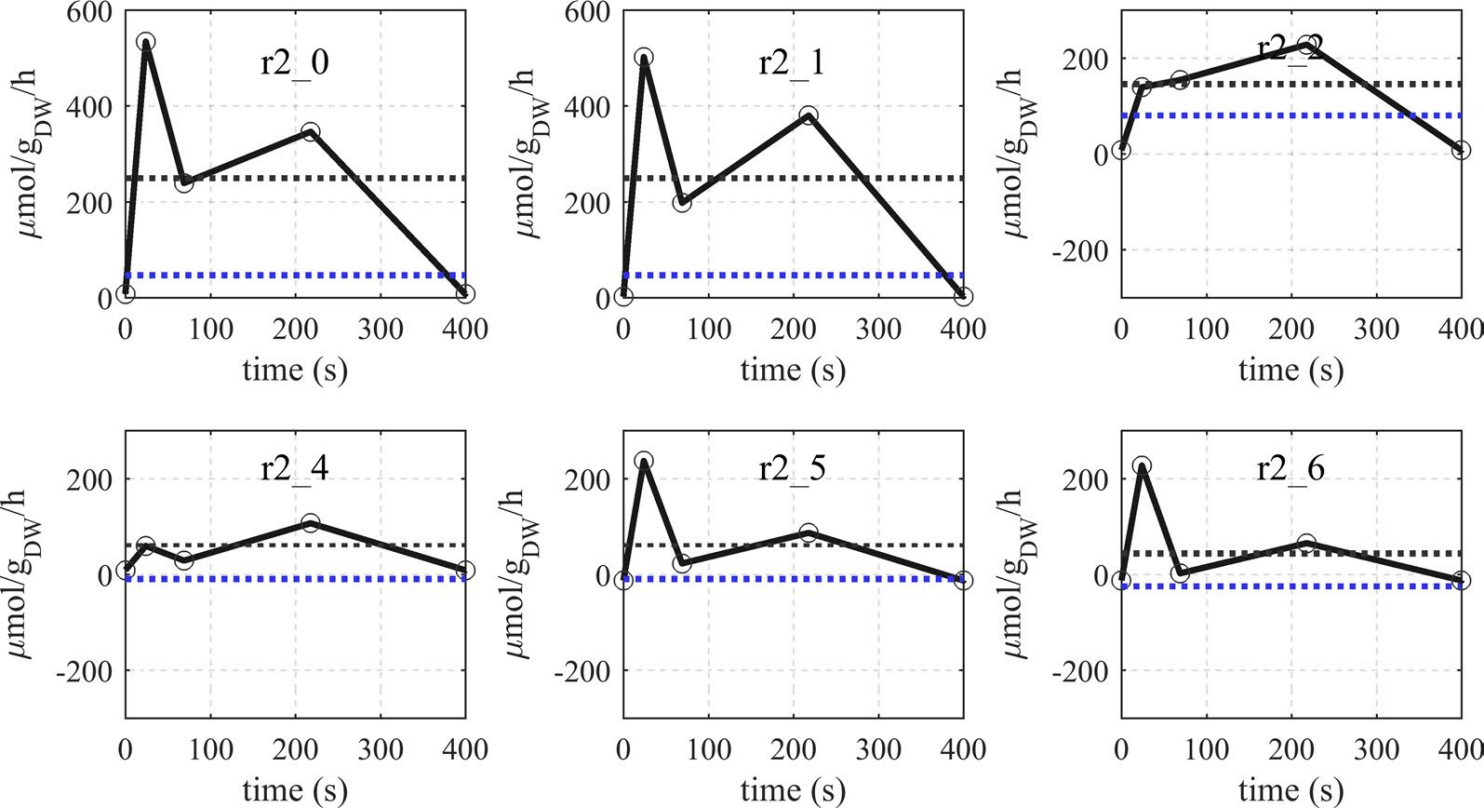
Dynamic fluxes of the pentose phosphate pathway during the feast/famine conditions. Horizontal lines represent: average flux over the cycle (black) and steady-state flux a D = 0.1 h^−1^ (blue)

The metabolic flux of the pentose phosphate pathway is different to that of the glycolysis (Fig. 6). After a rapid increase with the feeding phase, the flux decreased until about 70 s after which it increased again showing a second but lower peak at 220 s with the exception of ribulose-5-phosphate isomerase (reaction r2_2) and transketolase (reaction r2_4) where a higher peaks were obtained. Fluxes of the oxidative PPP increased rapidly from almost zero up to about 534 $\upmu {\text{mol g}}_{\text{DW}}^{ - 1} {\text{h}}^{ - 1}$. Although ribulose-5-phosphate-3-epimerase reaction (reaction r2_3) seemed to follow the described behavior, the slightly negative flux in the beginning (end) of the cycle suggests that this reaction operated backwards to feed into the R5P pools when substrate is limited. It is noteworthy to mention that this reaction also seems to run backwards during the reference steady-state (D = 0.1 h^−1^). Similar behavior was observed for transaldolase (reaction r2_5) and the E4P-consuming transketolase (reaction r2_6).

### Dynamic flux estimation of storage metabolism synthesis and degradation

**Fig. 7:**
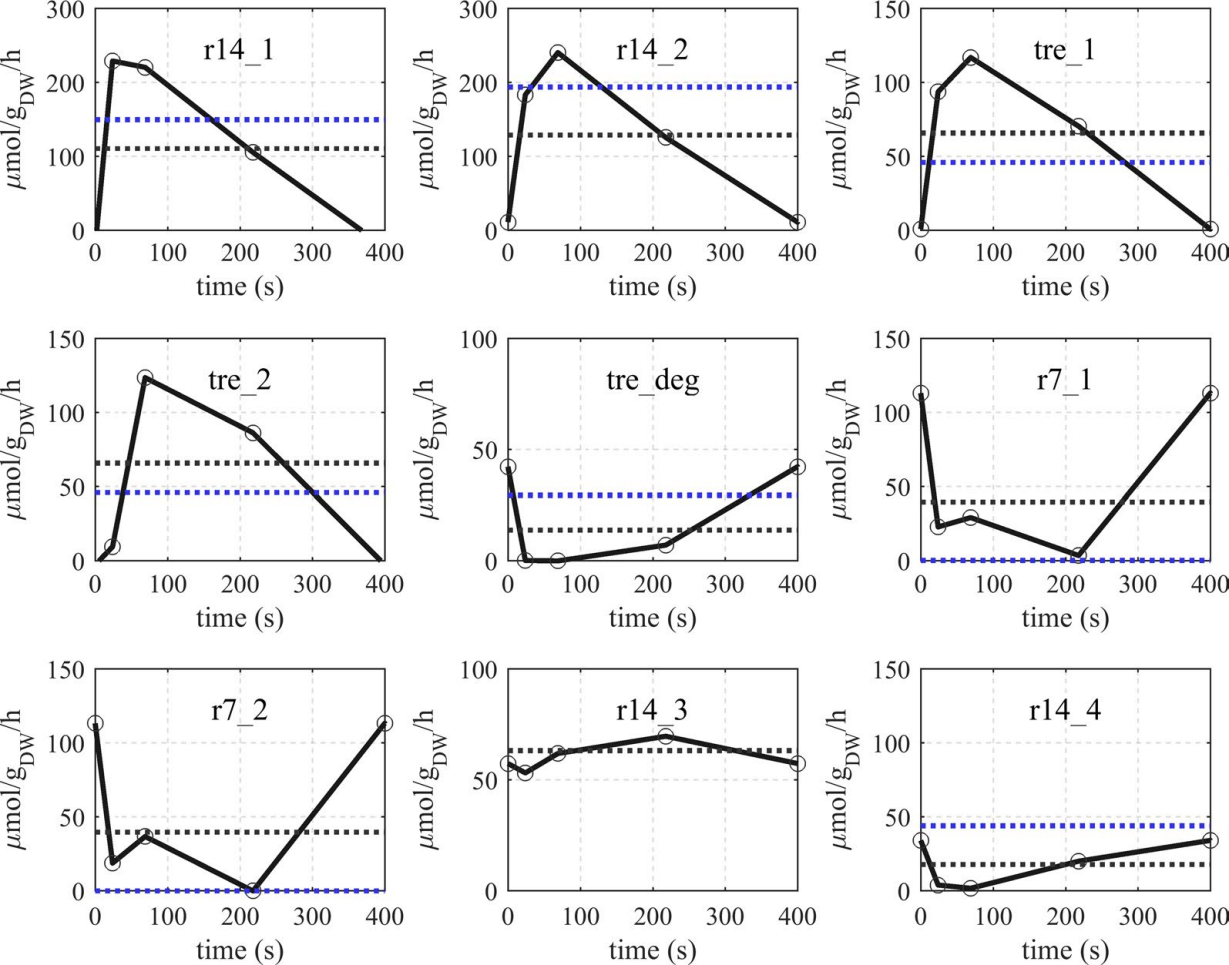
Estimated dynamic flux of storage synthesis and degradation pathways during the feast/famine regime. Horizontal lines represent: average flux over the cycle (black) and steady-state flux a D = 0.1 h^−1^ (blue)

Although exhibiting some delay, reactions of the trehalose synthesis (reactions tre_1 and tre_2) were activated by the glucose perturbation (Fig. 7). Especially, the flux of trehalose-6-phosphate synthesis increased from close to 0 to about 117 $\upmu {\text{mol g}}_{\text{DW}}^{ - 1} {\text{h}}^{ - 1}$ at 70 s after the glucose feeding. The significant delay in reaching the maximal flux, as compared with other reactions around the G6P branch point, suggests the presence of a putative post-translational regulation which was already speculated based on the concentration profile [8]. Correspondingly, trehalose synthesis seems to depend on its precursor, T6P. Considering the average fluxes of both, trehalose synthesis and degradation (66 and 14 $\upmu {\text{mol g}}_{\text{DW}}^{ - 1} {\text{h}}^{ - 1}$, respectively), a net trehalose export flux of 40 $\upmu {\text{mol g}}_{\text{DW}}^{ - 1} {\text{h}}^{ - 1}$ is expected in order for the trehalose concentration to remain nearly constant over the entire cycle.

The intracellular degradation of trehalose (reaction tre_deg) decreased directly after the glucose addition. The extracellular degradation of trehalose (reaction r7_2) seemed to be active in phases with low glucose availability (Fig. 7). Clearly, with lower glucose concentration, the thermodynamic driving force increases and influences the extracellular degradation flux.

Coming back to the initial hypothesis for the sources of the observed glucose de-enrichment, the model contained (Fig. 7):

1. A glucose recycle in which trehalose is first exported (reaction r_7_1) and subsequently degraded in the extracellular space (reaction r_7_2),
2. A glycogen breakdown followed by the export of the resulting glucose (reactions r14_4). Upon inspection of both routes, it can be observed that extracellular trehalose degradation accounts for about 40%, while glycogen degradation contributes 60% of the unlabeled glucose. Trehalose export and degradation (when other carbon sources were depleted) has been observed previously by [26] under batch conditions. The extracellular localization is in agreement with the observation that the acid trehalase (Ath1p) is localized in both, the periplasm and the vacuole [29] with an optimal pH between 4.5 and 5.0.

The synthesis of glycogen followed a different pattern to that of trehalose during the feast/famine. While the trehalose was mostly synthesized during high glucose availability, glycogen synthesis from UDPG (reaction r_14_3) showed a minimum of 53 $\upmu {\text{mol g}}_{\text{DW}}^{ - 1} {\text{h}}^{ - 1}$ at about 24 s and reached its maximal value at 220 s when glucose was already limiting. The degradation of glycogen is assumed to result in G1P and glucose (reaction r_14_4) as reported by [24]. Please note, that intracellular glucose was not included in our model and the simplification of a direct export of glucose was used in the model, i.e. glycogen degradation produces extracellular glucose and G1P in equal quantities. The estimated degradation flux (r_14_4) was close to zero during the first half of the cycle, which is in agreement with previous reports indicating that this reaction is inhibited by G6P and UDPG [7, 24].

### Putative trehalose kinase

The measured T6P enrichment pattern (Fig. 4), showing labeling enrichments below that of the precursors G1P and UDPG, requires an additional unlabeled inflow reaction. The estimated flux of a putative mechanism producing T6P from trehalose suggests activity in the second half of the cycle with fluxes increasing from 0 at 24 s to about 39 and 18 $\upmu {\text{mol g}}_{\text{DW}}^{ - 1} {\text{h}}^{ - 1}$ between 220 and 400 s, respectively. This backward flux accounted for about 30% of the net flux. Without such a putative mechanism the T6P enrichment profile could not be reproduced. Trehalose phosphatase is an irreversible reaction, therefore, this putative mechanism could involve an ATP-dependent trehalose phosphorylation reaction. While there is no trehalose kinase reported for *S. cerevisiae* there could be side-activities of another, related kinase.

### Thermodynamic considerations of fluxes during the feast/famine cycle

Metabolic reaction directions follow the thermodynamic driving forces (TDF) [8, 14]. Especially, reactions operating close the thermodynamic equilibrium could change direction during the feast/famine regime. We compared the results from the ^13^C flux estimation to the thermodynamic driving force (1-Q/*K*_*eq*_) of some reactions operating near equilibrium.

**Fig. 8:**
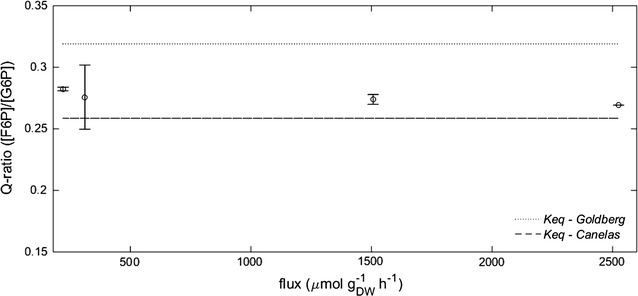
Action mass ratio (Q) for the PGI reaction. Two different values for *Keq* reported in the literature are shown: dashed line [14] and dotted line [30]

Contrary to the suggestion of a negative TDF for the PGI reaction [8], we found that the dynamic net flux (reaction r1_3) was always positive during the cycle suggesting a forward operation, although it was close to zero towards the end of the cycle. Note that the predicted flux direction (Fig. 8) depends on the assumed *(*in vivo*) K*_*eq*_. There may be changes in TDF, especially when changing conditions are expected (e.g., changes in intracellular pH). Here, we found that the mass action ratio (Q-ratio) of the PGI reaction tends to keep constant for all the calculated fluxes, as it is usual for reactions close to equilibrium. However, there is a slight tendency to decrease when the flux is higher. While the TDF would approach a zero value in case of a *K*_*eq*_ as reported by [14], the TDF would be maximal and positive in case of the value reported by [30]. Maximal flux for PGI was observed at about 15 s, which would be in agreement with the value reported by [30].

The metabolic flux estimation suggests a change in flux direction in PGM (r14_1) from G1P synthesis to G6P production towards the end of the cycle. Our results are in agreement with expectations from [8] since the dynamic flux of reaction r14_1 in Fig. 7 becomes slightly negative by the end of the cycle, indicating that this reaction runs towards the production of G6P. Fluxes for RPI (r2_2) and GAPDH&PGK (r1_7) are in agreement with thermodynamic considerations when based solely on concentration measurements.

### Comparison of steady-state and feast/famine conditions

To compare the metabolic activity with steady-state observations, the average fluxes during the cycle were calculated. The average flux of the glucose uptake (r1_1) equals to 1253 $\upmu {\text{mol g}}_{\text{DW}}^{ - 1} {\text{h}}^{ - 1}$ , which is higher than the glucose feeding rate. The here estimated uptake rate takes into account the glucose recycle produced from the extracellular trehalose degradation (r7_2), and the export of glucose produced from glycogen degradation (r14_4).

**Fig. 9:**
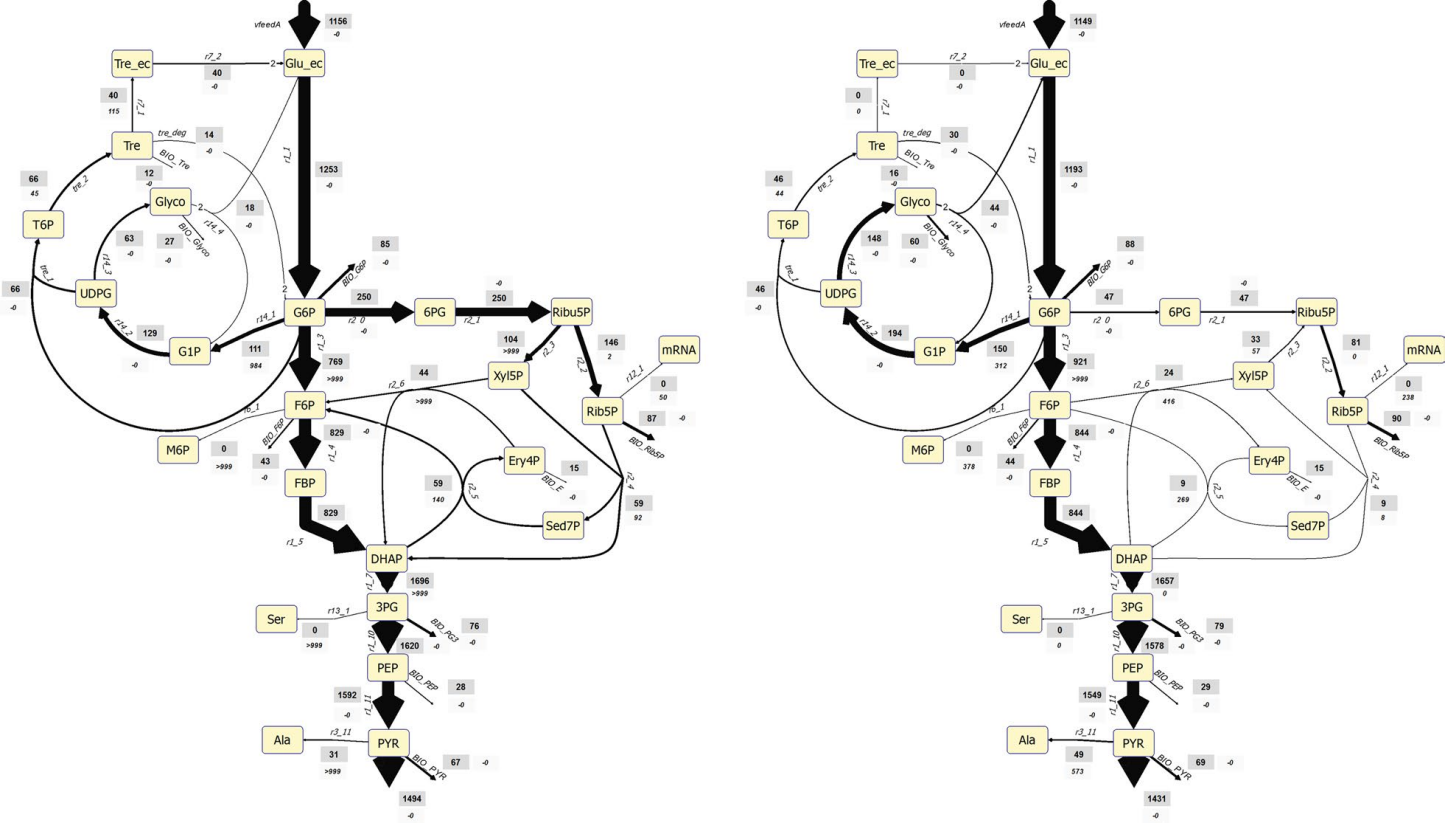
Comparison of flux distribution, on the left the average over the feast/famine cycle, on the right the reference steady-state (adapted from [32]). All values are given in µmol gDW-1 h^−1^

The imported glucose is then distributed over different pathways at the level of G6P (Fig. 9). Based on the average fluxes we found a flux distribution around the G6P branch point that agrees with previous reports [24] but is different to the reference steady-state. Remarkably, during the feast/famine the relative flux towards PPP increased from 3.8 to 20% while correspondingly the flux to glycolysis decreased from 74 to 61%. In contrast, the relative flux to the trehalose metabolism increased moderately from 4 to 5.2%.

In contrast to steady-state, under the dynamic feast/famine regime flux direction changes are observed. Especially, the PGM flux changes to the direction G1P → G6P between 364 s of the previous cycle to 2.5 s of the subsequent cycle. Similarly, a change in the flux direction of transaldolase (TA) and the F6P-producing transketolase (TK2) is observed, although only for very short periods. While during the steady-state TA run backwards (producing Sed7P) with a flux of about 9 $\upmu {\text{mol g}}_{\text{DW}}^{ - 1} {\text{h}}^{ - 1}$, under the feast/famine the flux was only reversed between (377 and 1.5 s) while the average forward flux (to F6P) was 59 $\upmu {\text{mol g}}_{\text{DW}}^{ - 1} {\text{h}}^{ - 1}$. Similarly, TK2 flux changed its direction during the feast famine towards the production of F6P. Thus under very low glucose availability, the pentose phosphate pathway feeds into glycolysis.

## Discussion

In this work we studied the dynamics of central carbon metabolism as well as trehalose and glycogen under repetitive perturbations, i.e. a block-wise feeding regime. The intracellular metabolites respond rapidly to the extracellular perturbations with up to ninefold change during the cycle. The perturbation led to a highly dynamic flux response, especially, the glucose uptake increased from 170 to 4788 $\upmu {\text{mol g}}_{\text{DW}}^{ - 1} {\text{h}}^{ - 1}$ in 24 s. Downstream glycolytic fluxes also exhibited fast changes ranging from 7- to 9-fold. A dramatic increase of nearly 90-fold in T6P synthesis was observed, indicating that storage metabolism is very sensitive to changes in the glycolytic flux and counterbalances rapid changes by diverting flux into large pools while at low glucose availability trehalose degradation is observed.

We found that in average about 15% of the consumed glucose is recycled, a significant fraction through trehalose in the intra- but also extracellular space. This was surprising, but in line with the reported trehalose Agt1p transporter [26] and a thermodynamically feasible extracellular degradation by acid trehalase [27]. This cycle can explain part of the observed de-enrichment of ^13^C in extracellular glucose. A further part of the de-enrichment originates from the estimated glycogen degradation flux.

Next to slightly reduced storage turnover, there were differences between the reference steady-state flux distribution and the average flux during the feast/famine regime:

(1) A fivefold increase in the oxidative pentose-phosphate flux. This higher flux generated a reversed flux of transaldolase and the F6P-producing transketolase reactions as compared to flux distributions at steady-state conditions.

Interestingly, during the short period of the cycle, some reactions do change direction. Especially, ribulose-phosphate-3-epimerase, transketolase and aldolase operated backwards towards the end of the cycle when glucose availability was low. Similarly, PGM switches from G6P consumption to G6P production. The observed concentration changes are proportional to the flux estimations, but in vivo *K*_*eq*_ seems to be still inconsistent in literature.

(2) Next to a de-enrichment in extracellular glucose, a decreasing ^13^C-profile was found for T6P that suggests a putative mechanism for trehalose phosphorylation. If this reaction was not considered, it was not possible to reproduce the experimental observations since the ^13^C-enrichment of G6P and UDPG remained high. The flux of this putative reaction was about 30% of the T6P-phosphatase flux and was estimated higher when glucose availability was low.

Although the accuracy of flux estimations in this work have not been evaluated statistically due to its inherent complexity, the fluxes reported here correspond to those with the least-squares that simultaneously fit both concentration and labeling enrichment measurements. Thus, small changes in flux values would cause ^13^C-labeling patterns to deviate dramatically. Nevertheless, the ability of the model and the estimated fluxes to reproduce most of the metabolic pools suggest that the method is robust and reliable. Deviations in labeling patterns of some pools could be expected due to missing reactions involving these pools or errors due to experimental conditions (e.g., sampling not fast to capture rapid changing conditions).

To obtain a more comprehensive understanding of the complete central carbon metabolism under dynamic conditions, a next step could be extending the present work to cover TCA cycle. This would demand more advance analytic and experimental techniques than the ones used here. Especially with relation to compartmentation of TCA cycle metabolites, a big challenge that can be resolved in the near future [31].

## Conclusions

In this work the successful implementation of an experimental platform plus a calculation methodology for estimating fluxes based on dynamic ^13^C labeling experiments shows that the metabolic response to changing substrate availability can unravel unreported metabolic behaviors.

The proposed approach was robust and it was possible to reproduce experimental data of most dynamic metabolite concentrations and dynamic ^13^C labeling patterns during multiple cycle perturbations. The approach can be applied to study metabolic responses to industrial conditions like substrate gradients but also changes in oxygen availability. While laborious, the approach can reveal intracellular cycles that can impact product yields and rates. In particular, upon strong changes in concentrations, some metabolic fluxes did not show strong responses indicating that the biological system is robust enough to withstand such changing environments. On the other hand, some reactions were found to change the direction of their metabolic flux as compared to steady-state fluxes. In addition, the observed dynamic behavior in glycogen and trehalose metabolism shows that at the beginning of the cycle, when substrate depletion has been reached, both glycogen and trehalose pools serve as a carbon source. This carbon utilization will proceed until a new carbon excess condition is present again.

Finally, emphasis has to be made on the fact that a feast/famine cultivation of *S. cerevisiae* exhibits a different metabolic behavior from both batch and continuous cultures, that is, the feast/famine regime does not necessarily resemble the features typically observed in chemostats or in batch. For instance, this can be noted in the no presence of ethanol and glycerol during periods of carbon excess when *S. cerevisiae* is grown under mild feast/famine conditions.

## Abbreviations

### Metabolites

2PG, 2-phosphoglycerate; 3PG, 3-phosphoglycerate; 6PG, 6-phospho gluconate; ALA, alanine; DHAP, dihydroxy acetone phosphate; E4P, erythrose-4-phosphate; F6P, fructose-6-phosphate; FBP, fructose-1,6-bis-phosphate; G1P, glucose-1-phosphate; G6P, glucose-6-phosphate; GAP, glyceraldehyde-3-phosphate; PEP, phospho-enol-pyruvate; PYR, pyruvate; Rib5P, ribose-5-phosphate; Ribu5P, ribulose-5-phosphate; SER, serine; S7P, sedoheptulose-7-phosphate; T6P, trehalose-6-phosphate; UDPG, UDP-glucose; UDP, uridine-5-diphosphate; UTP, uridine-5-triphosphate; X5P, xylulose-5-phosphate;

### Enzymes and/or reactions

ENO, phosphopyruvate hydratase; FBA, fructose-bisphosphate aldolase; G6PDH, glucose-6-phosphate dehydrogenase; GAPDH&PGK, glyceraldehyde-3-phosphate dehydrogenase + phosphoglycerate kinase; GPM, phosphoglycerate mutase; PFK, 6-phosphofructokinase; PGI, glucose-6-phosphate isomerase; PGM, phosphoglucomutase; PMI, mannose-6-phosphate isomerase; PYK, pyruvate kinase; RPE, ribulose-phosphate 3-epimerase; RPI, ribose-5-phosphate isomerase; TPP, trehalose- phosphatase; TPS, alpha,alpha-trehalose-phosphate synthase; TA, transaldolase; TK1, S7P-producing transketolase; TK2, F6P-producing transketolase.

### Other

CER, Carbon evolution rate; DO, dissolved oxygen; IDMS, Isotope dilution mass spectrometry; OUR, Oxygen uptake rate; PPP, pentose phosphate pathway; SRE, Stimulus response experiment.
